# Combined PET/CT-perfusion in patients with head and neck cancers might predict failure after radio-chemotherapy: a proof of concept study

**DOI:** 10.1186/s12880-015-0102-z

**Published:** 2015-12-29

**Authors:** Carsten Pietsch, Felipe de Galiza Barbosa, Martin W. Hüllner, Daniel T. Schmid, Stephan K. Haerle, Gerhard F. Huber, Gabriela Studer, Thomas F. Hany, Patrick Veit-Haibach

**Affiliations:** Department of Nuclear Nuclear Medicine, University Hospital Zurich, Ramistrasse 100, 8091 Zuerich, Switzerland; Department of Oto-Rhino-Laryngology-Head and Neck Surgery, University Hospital Zurich, Zurich, Switzerland; Department of Radiation Oncology, University Hospital Zurich, Zurich, Switzerland; Department of Diagnostic and Interventional Radiology, University Hospital Zurich, Zurich, Switzerland; University of Zurich, Zurich, Switzerland

**Keywords:** CT perfusion, PET/CT, Head and neck cancer, Chemo-radiotherapy

## Abstract

**Background:**

[18F]FDG-PET/CT imaging is broadly used in head and neck cancer (HNSCC) patients. CT perfusion (CTP) is known to provide information about angiogenesis and blood-flow characteristics in tumors. The aim of this study was to evaluate the potential relationship of FDG-parameters and CTP-parameters in HNSCC preand post-therapy and the potential prognostic value of a combined PET/CT with CTP.

**Methods:**

Thirteen patients with histologic proven HNSCC were prospectively included. All patients underwent a combined PET/CT with integrated CTP before and after therapy. Pre- and post-therapeutic data of CTP and PET of the tumors were compared. Differences were tested using Spearman’s rho test and Pearson’s correlation. A *p*-value of *p* <0.05 was considered statistically significant. Correlations were calculated using Pearson’s correlation. Bootstrap confidence intervals were calculated to test for additive confidence intervals.

**Results:**

Three patients died due to malignancy recurrence, ten patients were free of recurrence until the end of the follow-up period. Patients with recurrent disease had significantly higher initial CTP-values compared to the recurrence-free patients: BFpre 267.4 (171.2)ml/100 mg/min, BVpre 40.9 (8.4)ml/100 mg and MTTpre 8.2 (6.1)sec. No higher SUVs initially but significantly higher TLG compared to patients without recurrence were found. Post-therapeutic PET-values differed significantly between the two groups: SUVmaxpost 6.0 (3.2), SUVmeanpost 3.6 (2.0) and TLG 21751.7 (29794.0).

**Conclusion:**

In our proof of concept study, combined PET/CT with integrated CTP might show complementary prognostic data pre- and post chemo-radiotherapy. CTP may be used to predict local tumor recurrence, while FDGPET/CT is still needed for whole-body staging.

## Background

The diagnostic evaluation of head and neck cancer mainly relies on computed tomography (CT), magnetic resonance imaging (MRI) and [18F]Fluoro-2-deoxy-D-glucose-positron emission tomography/computed tomography (FDG-PET/CT). The main goal for work up of these tumors is local staging and detection of lymph node metastases as well as distant metastases. However, CT and MRI provide mainly morphological imaging while PET/CT provides a combination of metabolic information e.g. viability of the cell, tumor aggressiveness and proliferation in head and neck cancer and morphological information.

CT-Perfusion (CTP) is an imaging tool which provides functional information too, however, not primarily about glucose consumption but about perfusion behavior of a tumor [1–3]. It was shown that CTP has a positive correlation with microvessel density and can therefore partly reflect neo-angiogenesis in tumors. Furthermore, prognostic impact has been found in several studies evaluating CTP in head and neck cancer as well as in other tumor entities [1, 4–7].

Even if CTP and its potential use has been evaluated for several years, it has not been widely used in clinical routine so far. On the other hand, FDG-PET/CT is a broadly used imaging procedure for oncological staging, therapy follow-up and evaluation of prognosis in a wide variety of cancer as well as head and neck cancer [7–9].

There is currently insufficient information about the potential relationship of FDG-parameters [SUVmax, SUVmean, Tumor Lesion Glycolysis (TLG)] and CTP-parameters [Blood Flow (BF), Blood Volume (BV) and Mean Transit Time (MTT)] in head and neck cancer pre- and post-therapy and the potential impact of PET/CT-perfusion on how to select patients for the adequate therapeutic option (e.g. surgery/chemo-radiotherapy) [10, 11].

The aim of this proof of concept study was 1. to compare and to correlate CTP-parameters (Blood Flow (BF), Blood Volume (BV) and Mean Transit Time (MTT) and PET-parameters (SUVmax, SUVmean, Tumor Lesion Glycolysis (TLG) pre- and post chemo-radiotherapy in patients with squamous cell carcinoma in the head and neck (HNSCC) and 2. Evaluate the prognostic value of such a combined imaging procedure concerning recurrence and overall survival in these patients.

## Methods

### Patients

Overall 41 patients were prospectvely included in a study with PET/CT and integrated CT-perfusion. Of those, thirteen patients (mean age: 63 years; range: 44–78 years; one female, 12 male) received a PET/CT with CT-perfusion before and after therapy. The excluded patients hat either 1. only PET/CT without CT-perfusion or 2. were directly operated after staging or 3. received (salvage) surgery pre-or post radiochemotherapy during their cause of disease. Those patients were prospectively enrolled between 09/2008 and 02/2010. All patients had clinical suspicion for head and neck cancer and all were referred for routine primary staging and post-therapy follow up staging with FDG-PET/CT. The CTP-scan was integrated into the standard contrast-enhanced PET/CT-procedure. Patients were included consecutively, no further selection was applied. All patients had initially a biopsy proven squamous cell carcinoma: Nine carcinomas of the oropharynx (including three cancers of base of tongue and three of the tonsils), one oral cavity (floor of the mouth), one larynx carcinoma and two hypopharynx carcinomas. Eight of these patients had suspected lymph node metastases at the time of study enrollment. All patients received radio- and/or chemotherapy. Two patients additionally received salvage surgery (after radio- chemotherapy), nine patients received combined radio/chemotherapy (70Gy), one patient only chemotherapy and one patient only radiotherapy (70Gy). Different scientific aspects of the patients included in this study are already published in a different study [11]. Outcome of patients was assessed regularly based on local guidelines in our hospital. Patients with advanced head and neck cancer are seen clinically and with imaging after completion of therapy at 3, 6, 12 and 18 month or at occurrence of symptoms. If no recurrence is detected, patients are seen clinically every 2 years.

The study was performed in accordance with the regulations of the local institutional review board and ethics committee (Zurich Cantonal Ethics Committee). Written informed consent was obtained from all patients before the examination and enrolment into the study.

### Integrated FDG-PET/CT imaging

All data were acquired on a combined PET/CT in-line system (Discovery VCT, GE Healthcare, Milwaukee, WI, USA). These dedicated systems integrate a full-ring PET with a multislice helical 64-slice CT and permit the acquisition of co-registered CT and PET images in the same procedure. The patients fasted for at least 4 h prior to the examination. PET/CT imaging with integrated CTP was started 60 min after the injection of a standard dose of 300–340 MBq FDG. All patients received a non-enhanced CT examination which was acquired with the following parameters: 80 mA, 140 kV, 0.5-s tube rotation, 4.25-mm section thickness. The CT examinations were acquired during shallow breathing in the head and neck area, the thorax and the lower abdomen and during non-forced expiration in the upper abdomen. The CT examinations included the area from the head to the upper thighs. The PET emission examination was acquired with an acquisition time of 2 min per bed position after the low-dose CT examination. Images (15 cm axial field-of-view (FOV)/bed position) were reconstructed by using a standard fully 3D iterative algorithm (ordered subset expectation maximisation, OSEM: subsets, 28; iterations, 2; recon matrix, 128 × 128; overlap, nine slices). The selection of target lesions in the primary study was based on focal FDG uptake and the same region was selected in follow-up studies to CTP scan [11].

After target lesion definition, intravenous contrast injection was started by injecting a total dose of 70 mL contrast media (Ultravist 370, Bayer Schering Pharma, Germany). First, 40 mL of contrast media was applied at a flow of 5 mL/s via a cubital vein, and after a 5-s delay, the perfusion data were acquired for 50 s in the area of interest (1 s rotations time with one image/s, cine duration 50 s, eight slices, 5 mm slice thickness, 80 mA, 80 kV). Thus, an anatomical craniocaudal coverage of 4 cm was achieved for the perfusion examination of the target lesion. The first contrast media bolus injection was followed by a saline flush (20 mL, 5 mL/s). Directly after the perfusion imaging acquistion, a second bolus of another 30 mL of contrast media was applied at 4 ml/s followed by a saline flush of 30 mL to ensure full diagnostic (contrast-enhanced) ceCT data of the neck and thorax because all CTP examinations were fully integrated into the routine, clinical cePET/CT staging and re-staging examination [11].

### Image evaluation

#### CT-Perfusion evaluation

CTP data were evaluated by a dual-board-certified nuclear medicine physician/radiologist with 12 years of hybrid imaging experience, using a dedicated workstation (Advantage Workstation, 4.4, GE Healthcare, Milwaukee, WI, USA) with a commercially available perfusion software with a deconvolution method to calculate the perfusion values (CT-Perfusion 3, Body-Protocol) [11]. A freehand ROI defined within the margins of the target ENT-lesion (primary tumour) was placed in every slice of the perfusion volume (4 cm coverage, 5 mm slice thickness) in pre-therapeutic as well as post-therapeutic studies. In post-therapy scans the ROI was positioned in the area where tumor was seen in the previous scan. The calculated time enhancement curve and the parametric imaging maps for Blood Flow (BF, ml/100 mg tissue/min), Blood Volume (BV, ml/100 mg tissue) and Mean Transit Time (MTT, seconds) were automatically calculated by the software. The Δ-values (differences) between pre- and post-therapy scans were calculated afterwards. The permeability surface product was not calculated (short study protocol).

### Statistical analysis

The pre- and post-therapeutic values for CTP as well as pre- and post-therapeutic PET parameters were tested for differences using Spearman’s rho test and Pearson’s correlation. A p-value of *p* <0.05 was considered statistically significant. The correlation between all CT-perfusion data, the PET-data were and the Δ-values respectively were calculated using Pearson’s correlation. Secondary Spearman’s rho test was done for all data mentioned above. A *p*-value of *p* <0.05 was considered statistically significant. In addition the Bootstrap confidence intervals for both correlations were calculated to test for additive confidence intervals. All statistical analyses were performed using SPSS statistical software (Version 16.0.1, SPSS Inc., Chicago, Ill). To assess the prediction of local tumor recurrence by means of BVpre and SUVmaxpre, logistic regression models with stepwise forward entry were built for BVpre and SUVmax separately and using a combination of both with bootstrapping (1000 replications). The goodness-of-fit and the accuracy of the model were determined using the Hosmer-Lemeshow test and c-statistics (area under curve of the model).

## Results

### Patients

In all 13 patients malignant head and neck cancers was confirmed by histopathology. Staging was determined by clinical examination and imaging procedures in 11 patients. In the two patients additionally operated after chemo-radiotherapy (salvage surgery), clinical stage (see Table 1) was additionally confirmed by pathology after surgery. Of the 13 patients, three patients died due to recurrence of their malignancy during the follow up period, ten patients were free of recurrence until the end of the follow up period (of these one patient died of heart insufficiency 36 months after therapy recurrence free). The mean follow up period was 48.9 months with a minimum of 23 months and a maximum of 68 months. The mean time difference between the end of chemo-radiotherapy and the second scan was 10 weeks (min 6.7 and max 20 weeks). The mean survival of the patients with recurrence was 24 months and of the patients free of recurrence was 56.4 months.

**Table 1:** Clinical stage at enrolment

Clinical stage (*n* = 13)				
T-stage	2 × T1	7 × T2	2 × T3	2 × T4
N-Stage	4 × N0	1 × N1	2 × N2a	
			4 × N2b	
			2 × N2c	
M-stage	13 × M0 at enrolment			

### Pre- and post-therapeutic CTP-values and SUV-values of malignant lesions

**Fig. 1:**
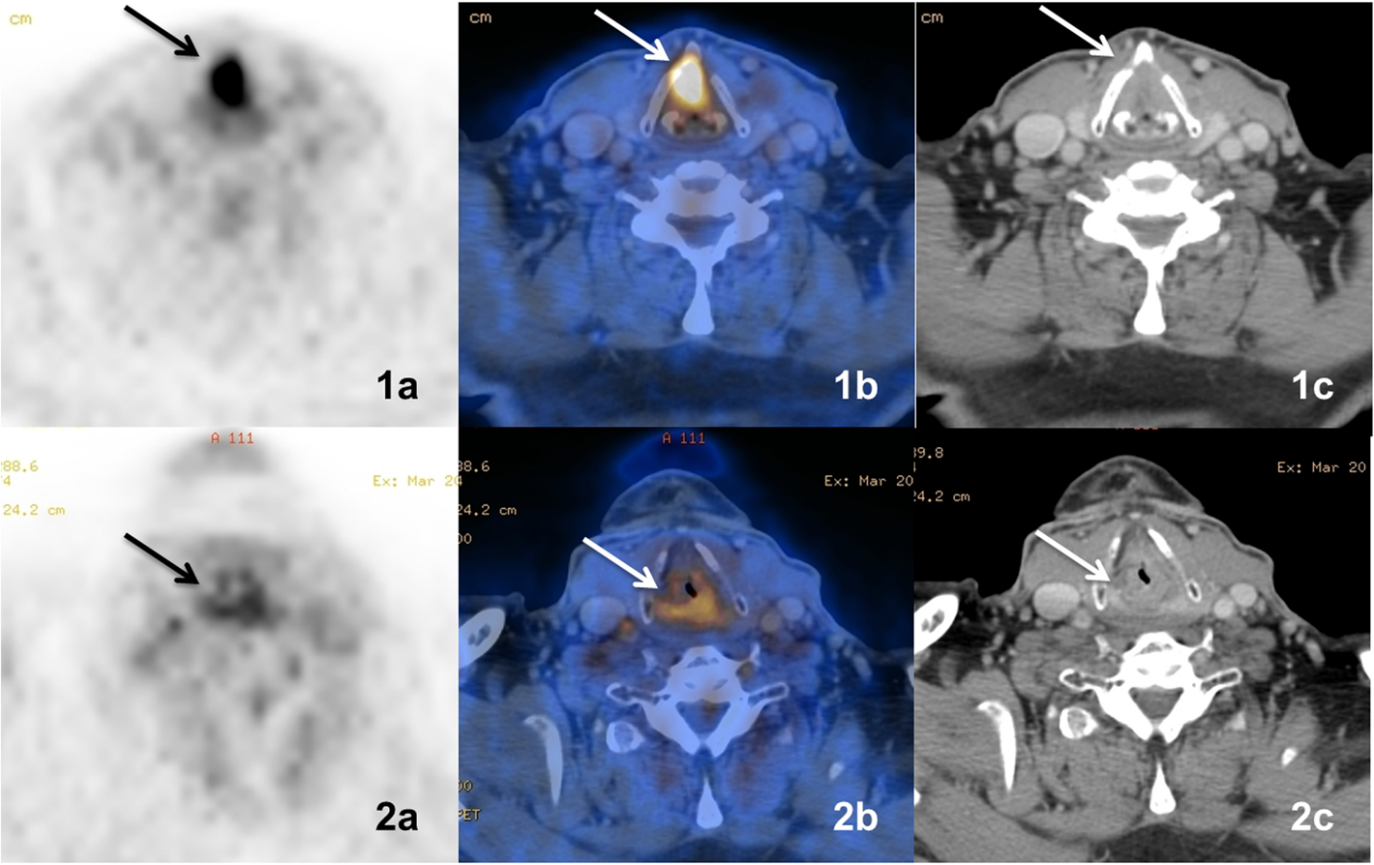
71 yo male with squamous cell carcinoma of the larynx. PET/CT. **1a**–**c** axial PET, PET/CT, CT with contrast. Pre-therapeutic scan showing the tumor (*arrow*). **2a**–**c** axial PET, PET/CT, CT with contrast. Post-therapeutic scan showing the residual reactive changes without tumor (*arrow*)

**Fig. 2:**
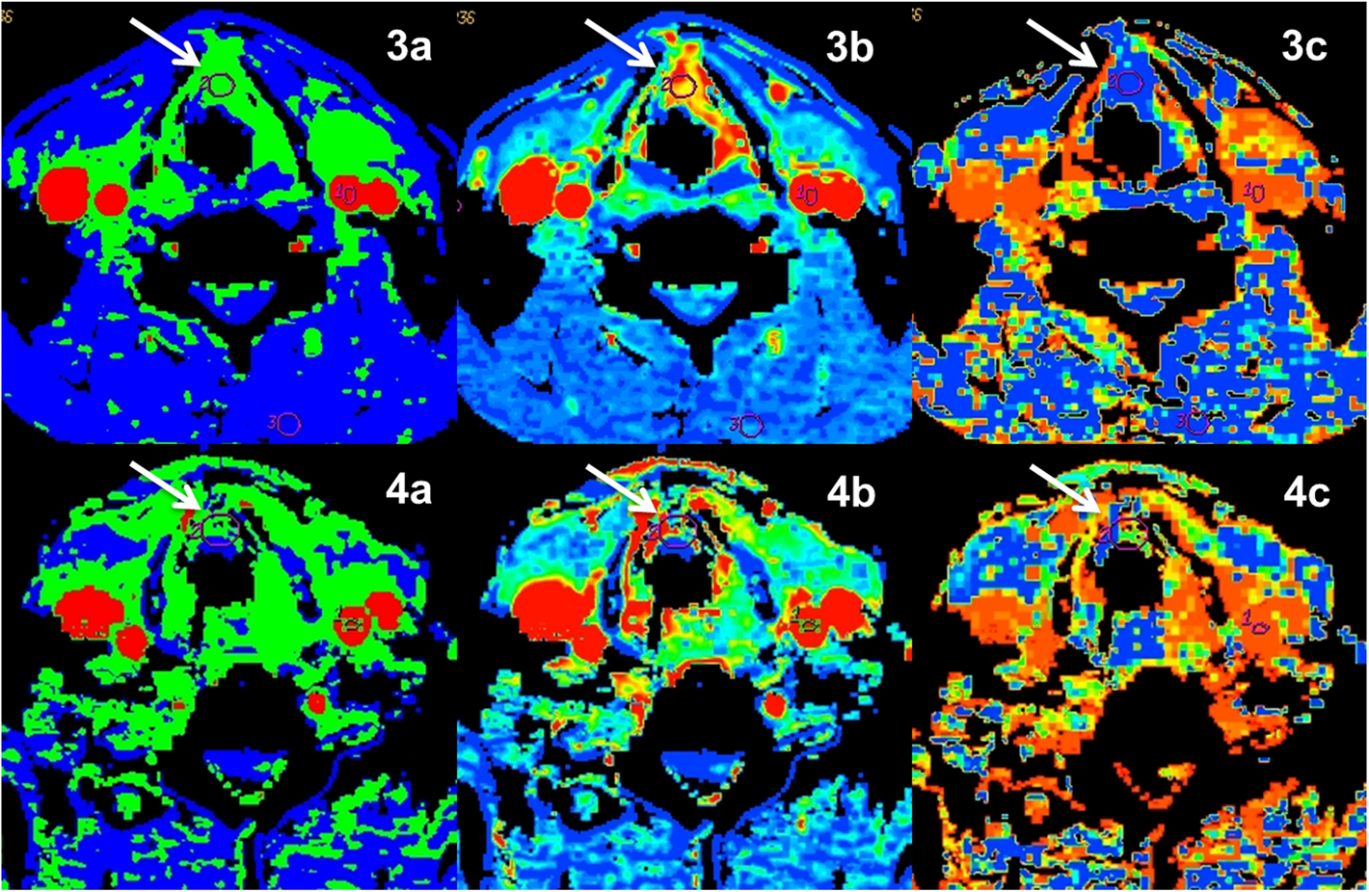
71 yo male with squamous cell carcinoma of the larynx. CTP. **1a**–**c**: axial Blood flow, Blood volume, Mean Transit Time. Pre-therapeutic scan showing the tumor (*arrow*). **2a**-**c** axial Blood flow, Blood volume, Mean Transit Time. Post-therapeutic scan showing the residual reactive changes without tumor (*arrow*)

First CTP-values and SUV-values, pre- versus postterapeutic including their change (Δ) were compared and all the mean values demonstrated in the Table 2 (see also Figs. 1 and 2). A comparison between the patients that died with recurrent disease and the recurrence free patients is shown in Table 3. Initial CTP-values were higher for the three patients that died during the follow up period but post-therapy CTP-values for those patients did not differ significantly from those without recurrence. Significant correlation was found between ΔBF and ΔBV (*p* <0.01).

**Table 2:** Pre- and Post-therapeutic mean CTP-values of all malignant lesions and consecutive differences

	Pre	SD^a^	Post	SD^a^	Δ (%)
BF (ml/100 mg/min)	193.4	133.6	69.4	31.8	−64.1
BV (ml/100 mg)	15.9	22.2	5.3	1.6	−66.7
MTT (sec.)	6.1	4.9	7.5	2.9	−23.3
SUVmax	9.4	4.7	3.8	1.6	−59.3
SUVmean	5.8	3.4	2.4	0.9	−58.9
TLG	61.3 × 10^3^	64.7 × 10^3^	27.9 × 10^3^	18.7 × 10^3^	−54.4

Significant correlations were only found between ΔBF and ΔBV (*p* <0.01). ^a^Standard deviation (SD)

**Table 3:** Comparison between Pre- and Posttherapeutic CTP-values in patients with recurrence and recurrence free patients

		Pre	SD^b^	Post	SD^b^	Δ (%)
BF (ml/100 mg/min)	Disease-free	171.2	123.7	70.2	34.11	−59.0
	Recurrence	267.4	161.7	67.1	22.4	−74.9
BV (ml/100 mg)	Disease-free	8.4	7.4	4.7	1.7	−34.9
	Recurrence	40.9	39.2	5.3	0.7	−88.4
MTT (sec.)	Disease-free	6.1	5.0	7.4	2.6	35.9
	Recurrence	8.2	3.5	7.8	3.9	−4.5
SUVmax	Disease-free	9.3	4.0	3.2^a^	1.3	−65.5^a^
	Recurrence	10.1	4.7	6.0^a^	1.6	−40.1^a^
SUVmean	Disease-free	5.7	3.0	2.0^a^	0.8	−64.7^a^
	Recurrence	6.2	3.0	3.6^a^	1.2	−41.4^a^
TLG	Disease-free	57.8 × 10^3a^	66.6 × 10^3^	29.8 × 10^3a^	20.3 × 10^3^	−48.4^a^
	Recurrence	79.3 × 10^3a^	51.4 × 10^3^	21.8 × 10^3a^	5.8 × 10^3^	−70.3^a^

Significant differences are marked with ^a^. Significant differences: TLG differed significantly in the pre-therapeutic scans. The Δ for SUVmax/mean and TLG differed significantly between patients with recurrence and those recurrence free. The post-therapeutic values for SUVmax/mean and TLG differed significantly between the patients that died and the recurrence free. Significant correlations: ΔSUVmax and ΔTLG (*p* <0.01) and ΔSUVmax and ΔSUVmean (*p* <0.05). ^b^Standard deviation (SD)

SUVmaxpre and SUVmeanpre showed no significant difference between recurrence free patients and the patients who died, however the pre-therapeutic TLG of recurrence free patients and the patients who died differed significantly.

There were significant correlations among ΔSUVmax and ΔTLG (*p* <0.01) and between ΔSUVmax and ΔSUVmean (*p* <0.05). The post-therapeutic values differed significantly between the patients that died and the recurrence free.

### Correlation of CT-perfusion data and PET-data

All pre- and posttherapeutic acquired CTP-data (BF, BV, MTT), the SUV-values (SUVmax, SUVmean, TLG) and the corresponding Δ-values were correlated (Tables 2 and 3).

No statistically significant correlation was found among the above mentioned corresponding CTP- and PET-values nor their Δ-values, respectively. In addition Bootstrap confidence intervals for these comparisons also showed no possible correlation within these intervals.

### Regression

**Fig. 3:**
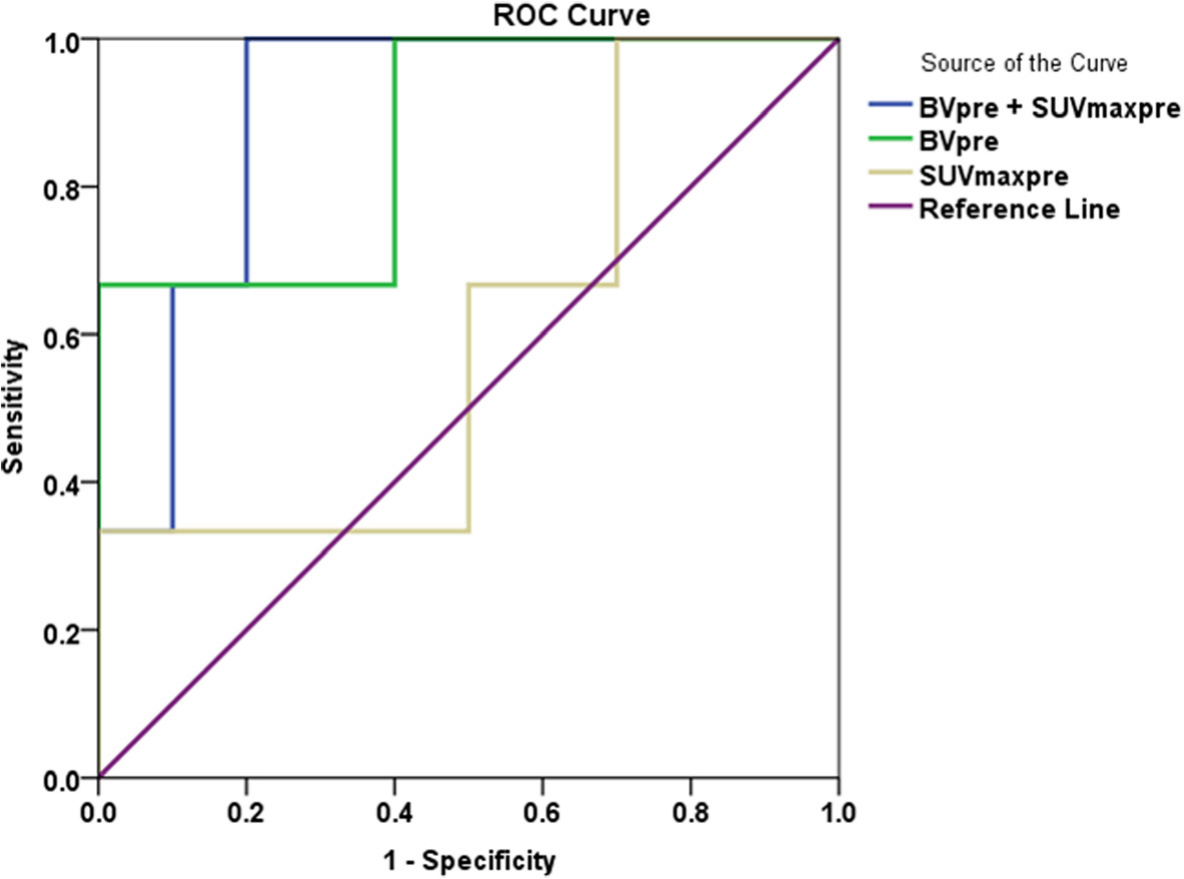
ROC (receiver operating characteristics) curves for BVpre and SUVmaxpre vs. local recurrence. BVpre: AUC = 0.867, 95 % CI, 0.616 – 1.000 (*p* = 0.063); SUVmaxpre: AUC = 0.600, 95 % CI, 0.210 – 0.990 (*p* = 0.612); BVpre combined with SUVmaxpre: AUC = 0.900, 95 % CI, 0.724 – 1.000 (*p* = 0.043). (AUC: area under curve)

Logistic regression models are demonstrated in Table 4, which summarizes the association between local recurrence and the different variables in the various model steps. Multivariate logistic regression showed that only SUVmaxpre slightly improved the initial model including BVpre alone (c statistics, 0.867 to 0.900). Higher SUVmaxpre was associated with a decrease in the odds for developing local recurrence if only BVpre was considered. This was associated with an increased *p*-value for BVpre. BVpre was therefore a significant independent predictor (*p* = 0.003) of local recurrence, with correct prediction in all three patients. Higher BVpre was associated with an increase in the odds for developing local recurrence if only SUVmaxpre was considered. SUVmaxpre was found to be a borderline significant independent predictor of local recurrence (*p* = 0.040) (Fig. 3).

**Table 4:** Logistic regression analyses for the prediction of local recurrence

	Coefficient	SE	β estimate	95 % CI	*p*	c statistics
*(a) Model for the probability of recurrence according to BVpre*						
BVpre	5.636	1.696	280.418	−0.685 – 6.162	0.08	0.867
*(b) Model for the probability of recurrence according to SUVmax*						
SUVmaxpre	0.036	4.603	1.036	−1.426 – 19.166	0.770	0.600
*(c) Model for the probability of recurrence according to BVpre and SUVmax*						
BVpre	1.992	0.144	7.329	−1.568 – 2.126	0.003	
SUVmaxpre	−3.048	1.447	0.047	−7.379 – -0.384	0.040	0.900

*SE* Standard error

*CI* confidence interval

C-statistics: accuracy of chosen statistical model

β estimate: resulting from a regression analysis hat have been standardized

## Discussion

The application and usefulness of CTP and PET/CT in staging and restaging of head and neck cancer, has been described before. However, to our knowledge there is no study evaluating the benefits of combined CTP and FDG-PET/CT in patients pre- and post radiochemotherapy in head and neck squamous cell carcinoma (HNSCC).

### Current CTP literature

It is already known that elevated blood flow on CTP is correlated with angiogenesis, microvessel density (MVD) and increased tumor vascularity, and is has been found to be correlated with tumor recurrence and the tumor’s ability to metastasize also in HNSCC [6, 7, 12]. Several studies also investigated the ability of CTP to predict therapy success in HNSCC and it was found that patients with decreased perfusion showed higher local recurrences [13] and higher perfusion (BV and BF) correlated with improved chemotherapy response [14]. Accordingly, patients with higher pre-treatment BF showed higher rates of locoregional tumor control [14–16]. One of the reasons for the ability of prediction of tumor outcome is assumed to be related to the fact that higher MVD leads to a higher oxygenation of tumor tissue. As oxygen is a known radiosensitizer, higher levels of oxygenation might lead to higher response rates, especially to radiation therapy but also concerning the effects of chemotherapy. This might be one of the reasons why HNSCC with high perfusion seem to profit from radiochemotherapy despite their worse prognosis.

### Current PET/CT literature

FDG-PET/CT is well established and widely used in staging and re-staging of solid tumors, not only in HNSCC but in a large variety of other tumors as well [10, 11, 17, 18]. It is a well understood pathophysiological concept that tumor cell metabolism correlates in different ways with the uptake of FDG (expressed by SUVmax). Hence, an elevated SUV in tumors might represent the expression of different intracellular pathways. This need for glucose in growing tumors can be based on increased glycolysis, increase of cell proliferation, increased synthesis rate and/or activation of different oncogenic pathways. It is also known that this demand for energy is also related to the production of acids, which are produced to give the tumor the ability to invade surrounding tissues [11]. PET/CT has been used for several years now for radiotherapy planning as well as for evaluation and prediction of recurrence [19]. However, there are partly conflicting results: while several authors concluded that PET/CT is an accurate tool to assess response after primary treatment of head and neck cancer and it is also a potential predictor of outcome after therapy, studies also recommended close follow-up with PET/CT to detect local failures [17, 20]. One possible reason is given by the study of Vainshtein and co-workers who found very low sensitivies and positive predictive value (PPV), but high negative predictive value (NPV) for the prediction of loco-regional failure after radiochemotherapy [18].

### CTP and PET/CT

Several studies have shown a good correlation between BF and SUVmax, respectively SUVmean in primary staging of HNSCC (as well as other tumors) [8, 12, 16, 21–26]. But there is also literature suggesting an inverse correlation between SUVmax and BF in HNSCC due to anaerob glycolysis [27]. Thus, although FDG-uptake can be considered as an additional marker for tumor characterisation, it might not be able to differentiate which of the above mentioned pathways is the predominant one. In those cases, the integrated CTP might be helpful.

In our study we measured initially high CTP values (BF, BV) during staging of tumor tissue as a possible marker for high MVD and tumor oxygenation. Interestingly in our study, those patients with recurrent disease had higher initial CTP-values as a possible marker for a more aggressive disease as compared to the recurrence-free patients. However, they did not show higher SUV’s initially but significantly higher TLG compared to patient without recurrence. After chemo-radiotherapy the CTP-values in these patients decreased to levels not significantly different from those patients without recurrent disease. This is partly contradictive to the studies discussed above where in patients with initially higher CT-perfusion values a favourable outcome was reported [28]. However, on the other side our results are in line with studies discussed in the overview from Preda and co-workers where CTP-parameters showed significant correlation with therapy response.

One difference might be that several of the above discussed manuscript reported about patients with radiotherapy, while patients in our study mainly received chemo-radiotherapy. Another reason which has to be acknowledged is the relatively low number of patients – in our study as well as in discussed manuscripts. We also found no significant correlation between the metabolic activity measured by SUVmax, SUVmean and TLG with the CTP-values. Their Δ-values after chemo-radiotherapy were not correlated, too.

On the other side, patients who died during follow-up based on their tumor recurrence had higher SUV-values after therapy in their follow-up scan and thus, indicating possible residual tumor. This is at least partly comparable to the aforementioned studies about PET/CT in follow-up of head and neck cancer after therapy where the metabolic values were found to be accurate and helpful for detection and prediction of recurrence.

In addition, our study showed that by analysing the pretherapeutic BV, a prediction of posttherapeutic local recurrence might be possible. SUVmaxpre was also able to predict local recurrence, however with borderline significance. Both parameters showed opposite tendencies, with higher BVpre and lower SUVmaxpre being associated with the development of local recurrence.

As there was no correlation between CTP- and FDG-PET/CT-values it seems that PET/CT with integrated CTP is able to measure two independent factors of tumor characteristics: on one hand the pre-therapy CTP-values can be considered a marker for tumor MVD and the corresponding level of aggressiveness and expected response to chemo-radiotherapy and overall survival. On the other hand the post-therapy PET-values may identify and re-assure the possible failures so that in those patients might get additional or prolonged therapy and/or more close follow-up. For such a diagnostic/prognostic and therapy evaluation work-up PET/CT with integrated CTP might provide complimentary data in one single examination.

### Limitations

We only have a small number of patients and thus, the results reported here can only serve as a proof of concept and as an indicator of the value of such a combined imaging approach. Additionally, the post-therapeutic PET/CT was not always done at the same time point in all patients. Lastly, the majority, but not all patients received the same treatment.

## Conclusion

Combined PET/CT with integrated CTP might show complementary prognostic data pre- and post-chemo-radiotherapy. CTP may be used to predict local tumor recurrence, while FDG-PET/CT is still needed for whole-body staging and might see residual tumours. Thus, this integrated approach might be used for the diagnostic and prognostic work-up in head and neck cancer.

## Abbreviations

HNSCC: head and neck squamous cell carcinoma
CTP: computed tomography perfusion
SUV: standardized uptake value
SUVmax: maximum standardized uptake value
SUVmean: mean standardized uptake value
TLG: tumor lesion glycolysis
BV: blood volume
BF: blood flow
CT: Computed tomography
ce – e.g. ceCT: contrast-enhanced
MRI: Magnetic resonance imaging
FDG-PET/CT: [18F]Fluoro-2-deoxy-D-glucose-positron emission tomography/computed tomography
CTP: CT-Perfusion
MTT: Mean Transit Time
ENT: Ear nose and throat
EGFR: Epidermal growth factor receptor
MVD: Microvessel density
Δ: delta or difference
pre – e.g. pre-treatment: previous
post – e.g. post-treatment: and post/after
